# Dry-Process Polymer Toughening of High-Modulus Asphalt Mixtures: Rheological Response and Competitive Absorption

**DOI:** 10.3390/polym18212777

**Published:** 2026-11-07

**Authors:** Kaixin Guo, Peixing Yang, Zhaohui Min, Jinxiang Hong

**Affiliations:** 1 School of Transportation, Southeast University, Nanjing 211189, China; kaixin_guo03@163.com; 2 Intelligent Transportation System Research Center, Southeast University, Nanjing 211189, China; 3 Jiangsu Sobute New Material Co., Ltd., Nanjing 211103, China; hongjinxiang@cnjsjk.cn

**Keywords:** high-modulus asphalt mixture, dry-process conditioning, co-swelling behavior, stress relaxation, multi-scale evaluation

## Abstract

High-modulus asphalt mixtures (HMAMs) achieve high stiffness but exhibit insufficient resistance to low-temperature cracking. To balance high- and low-temperature performance, HMAMs containing 0.3%, 0.6%, and 1.0% high-modulus modifier (HMM) were toughened with a styrene–butadiene-based polymer (SBP) through a dry process. The toughening behavior and mechanisms were investigated at different SBP dosages (0.2%, 0.4%, and 0.6%) and conditioning times using mixture tests, binder rheological characterization, individual and co-swelling tests, and molecular dynamics simulations. Increasing HMM content substantially improved dynamic modulus and rutting resistance but continuously reduced low-temperature bending strain. At 0.4%, SBP provided favorable strength–deformation compatibility, increasing bending strain by 76.1%, 75.3%, and 63.2% in the three HMM systems, respectively. The optimal conditioning time extended from 30 min (0.3% HMM) to 60 min (0.6% and 1.0% HMM). The 0.6% HMM–0.4% SBP combination exhibited the most balanced overall performance. Rheological results showed that HMM enhanced high-temperature elasticity, whereas SBP reduced low-temperature creep stiffness and improved stress relaxation. With limited availability of light fractions, increasing HMM from 0.3% to 1.0% reduced the swelling ratio of SBP from 114.87% to 62.30%. Molecular simulations further showed that HMM–light fraction interactions increased, whereas the magnitude of the SBP–light fraction interaction energy decreased by 13.2%. These findings suggest that competition for limited light fractions may contribute to the HMM-dependent toughening response, providing a basis for further investigation of material composition and conditioning requirements.

## 1. Introduction

With increasingly heavy, channelized traffic loads and frequent extreme temperatures, asphalt pavements require sufficient structural capacity, environmental adaptability, and long-term durability. High-modulus asphalt mixtures (HMAMs) are typically produced using hard-grade asphalt or by directly adding a high-modulus modifier (HMM) to increase stiffness, reduce traffic-induced structural strain, and improve rutting resistance; consequently, they are increasingly used in the base course, middle surface course, and lower surface course of heavy-duty pavements [1,2,3]. However, high-modulus modification may compromise the deformation adaptability of the mixture [4,5]. Furthermore, such modification alters the viscoelastic response and temperature sensitivity of asphalt binders, thereby affecting stress relaxation and crack propagation in the mixture [6,7]. Therefore, HMAM design must balance high stiffness and rutting resistance with deformation adaptability across a wide temperature range [8,9].

This balance is particularly critical at low temperatures, where high mixture stiffness coupled with insufficient binder stress relaxation accelerates thermal stress accumulation and increases pavement cracking risks [10]. Because low-temperature cracking is jointly affected by binder thermal contraction and stress relaxation, mixture structural constraints, and ambient temperature fluctuations [11], single-scale performance indicators cannot fully characterize this behavior; instead, an integrated evaluation of binder rheological responses and mixture failure characteristics is required [12,13]. To mitigate the trade-off between stiffness and low-temperature cracking resistance in HMAMs, fiber reinforcement and polymer modification are commonly employed. Although fibers primarily improve cracking resistance through physical bridging and reinforcement at the mixture scale, they fail to directly remedy the inadequate stress relaxation of stiff binders and often increase compaction resistance and may lead to a reduction in dynamic modulus [14,15]. Conversely, because the low-temperature embrittlement caused by high-modulus modification is closely related to insufficient binder relaxation, polymers can directly adjust the viscoelastic response and elastic recovery capacity of the matrix [16,17]. Nevertheless, the actual toughening effect of polymers depends heavily on their dispersion and conditioning within the asphalt; thus, the incorporation method and processing parameters are critical to modification efficacy.

Yan et al. [18] reported that conventional wet processes typically utilize high-temperature shearing to predisperse polymers in asphalt, leading to increased energy consumption and potential phase separation during storage. By contrast, the dry process introduces polymers directly into heated aggregates or mixtures, reducing preprocessing requirements and reliance on specialized equipment [19,20]. Recent studies demonstrate that well-designed dry-process polymer systems improve the high-temperature, low-temperature, and fatigue performance of mixtures. However, this modification efficacy remains highly sensitive to material composition and processing conditions [21,22]. Insufficient polymer dosages yield limited toughening, and excessive additions do not guarantee continuous performance improvements. Thus, determining the optimal polymer dosage is essential for dry-process polymer systems [23,24]. Unlike polymers predispersed by shearing in the wet process, dry-added polymers must progressively undergo physical dispersion, absorption, and swelling during mixing and subsequent high-temperature conditioning, while the polymer–asphalt interfacial structure evolves accordingly [25]. This process is highly sensitive to conditioning duration. Appropriately extending this time facilitates further absorption and swelling; however, excessive thermal treatment can accelerate base asphalt aging, offsetting further performance gains [26,27,28].

During dry-process conditioning, polymer absorption and swelling depend on the availability of mobile light fractions, particularly aromatic and other low-molecular-weight components that strongly influence polymer–asphalt compatibility and elastic network formation [29,30]. In systems containing both HMM and dry-added polymers, increasing HMM content may alter the availability of these fractions and thereby affect polymer development. However, it remains unclear whether variations in HMM content affect dosage-dependent toughening response and toughening efficiency or whether the high-temperature conditioning time required for sufficient toughening differs among HMM systems. This suggests a potential competition between HMM and polymers for limited mobile light fractions. Existing studies have generally examined HMMs and polymers separately, with limited attention to their coupled effects under identical material and mixture conditions. Moreover, this competitive absorption hypothesis has rarely been systematically investigated from macroscopic, mesoscopic, and microscopic perspectives.

The objective of this study was to evaluate the effectiveness of dry-process toughening with a styrene–butadiene-based polymer (SBP) in balancing the stiffness and low-temperature cracking resistance of HMAMs and to examine the potential role of competitive light-fraction absorption. Four specific tasks were undertaken: (1) quantify the performance trade-off induced by increasing HMM content; (2) identify suitable SBP dosages and conditioning times for different HMM contents; (3) clarify the rheological changes associated with SBP toughening; and (4) examine whether competition for limited light fractions could explain the HMM-dependent toughening response. This study clarifies how the toughening effect of SBP and the suitable conditioning time change with HMM content. The possible role of competitive light-fraction absorption is examined using swelling tests and molecular simulations. These findings support the selection of SBP dosage and conditioning time to balance low-temperature cracking resistance with stiffness, rutting resistance, and moisture stability.

## 2. Materials and Methods

The experimental program comprised five stages. First, mixtures containing 0%, 0.3%, 0.6%, and 1.0% HMM without SBP were evaluated as baseline systems. Second, SBP dosages of 0.2%, 0.4%, and 0.6% were compared using low-temperature bending tests after 30 min of conditioning. Third, the selected dosage of 0.4% SBP was used to evaluate conditioning times of 0, 15, 30, 60, and 120 min. Fourth, mixtures prepared using the selected dosage and conditioning times were assessed for dynamic modulus, rutting resistance, and moisture stability. Finally, binder rheological characterization, individual and co-swelling tests, residual light-oil compositional analysis, and molecular dynamics simulations were integrated to interpret the mixture performance results.

### 2.1. Materials

#### 2.1.1. Asphalt Binder and Aggregates

A 70-penetration-grade paving asphalt supplied by Sinopec Yanshan Petrochemical Company (Beijing, China) served as the base binder. Its basic properties are presented in Table 1 and satisfy the requirements of JTG F40. The coarse aggregate, fine aggregate, and mineral filler were supplied by Jiangsu Lubang Civil Engineering Technology Co., Ltd. (Changzhou, China). and met the ASTM D692, ASTM D1073, and ASTM D242 specifications for dense-graded asphalt mixtures, respectively.

#### 2.1.2. Modifiers

Two solid modifiers were used in this study. The high-modulus modifier (HMM), supplied by Jiangsu Zengguang New Materials Technology Co., Ltd. (Nantong, China), consisted of black polyolefin-based granules with a particle size of 2.0–3.0 mm. It had a density of 0.95 g/cm^3^, a melting range of 140–160 °C, and a melt flow rate (MFR) of 3.5 g/10 min (190 °C, 2.16 kg). The styrene–butadiene-based polymer (SBP), supplied by Sinopec Yanshan Petrochemical Company (Beijing, China), was used as the low-temperature toughening agent. It was a colorless, transparent powder with a styrene-to-butadiene mass ratio of 75:25, a density of 1.01 g/cm^3^, and an elongation at break ≥ 150%.

#### 2.1.3. Light Oil for Competitive Absorption

The light oil (LO), supplied by Sinopec Yanshan Petrochemical Company (Beijing, China), contained 74.13 wt% aromatics and 25.87 wt% saturates. LO was used as a simplified model system to examine modifier swelling and changes in residual aromatic and saturate contents under limited-supply conditions. The exclusion of resins and asphaltenes facilitated analysis of these selected fractions but omitted their potential contributions to component distribution, molecular mobility, and polymer–binder interactions. The model therefore represents a controlled light-fraction environment rather than the complete compositional and structural environment of asphalt binder.

### 2.2. Sample Preparation and Experimental Methods

#### 2.2.1. Mix Design and Preparation of Dry-Process HMAMs

The French continuous gradation for HMAC-16 was adopted, with a target air-void content of 4.0% ± 0.3% for all mixtures (Table 2). The optimum asphalt-to-aggregate ratio was determined as 5.8% using the Marshall method and engineering experience.

HMM contents of 0.3%, 0.6%, and 1.0% were investigated to examine the balance between stiffness and low-temperature cracking resistance. SBP contents of 0.2%, 0.4%, and 0.6% were evaluated to identify toughening benefits and potential adverse effects at higher dosages. Dosage selection considered bending strain and strength jointly, followed by conditioning-time evaluation and assessment of stiffness, rutting resistance, and moisture stability.

For dry-process preparation, aggregates were preheated to 190 °C and mixed sequentially with HMM, SBP, 160 °C base asphalt, and mineral filler, with 90 s of mixing after each addition. HMM and SBP dosages were expressed as percentages of dry aggregate mass. Baseline HMM mixtures were compacted without conditioning, whereas mixtures for SBP dosage optimization were conditioned at 160 °C for 30 min. For the conditioning-time study, the selected SBP dosage was fixed, and mixtures were conditioned for 0, 15, 30, 60, or 120 min at 160 °C. Four independently mixed replicate specimens were prepared for each group.

#### 2.2.2. Preparation of Model-Modified Binders

Based on the 5.8% asphalt-to-aggregate ratio, the dry-process HMM and SBP dosages were converted to equivalent dosages by mass of binder (Table 3). Model binders were prepared at these nominal modifier-to-asphalt mass ratios for comparative rheological characterization. The 70-penetration-grade base asphalt was heated to 175 °C and blended with HMM and SBP at 800 r/min for 30 min, followed by high-shear mixing at 4500 r/min and 190–200 °C for 60 min. All binders were subsequently conditioned at 160 °C for 60 min. HMM-only control binders were prepared using the same mixing and conditioning procedures but without SBP. Four independent batches were prepared for each formulation.

Model binders were prepared using high-shear mixing to facilitate modifier dispersion. Identical preparation conditions provided a consistent basis for comparing formulation-dependent rheological responses and interpreting mixture behavior. The converted dosages represent nominal mass ratios rather than equivalent dry-process conditions.

#### 2.2.3. Rheological and Physicochemical Characterization of Binders

**Figure 1:**
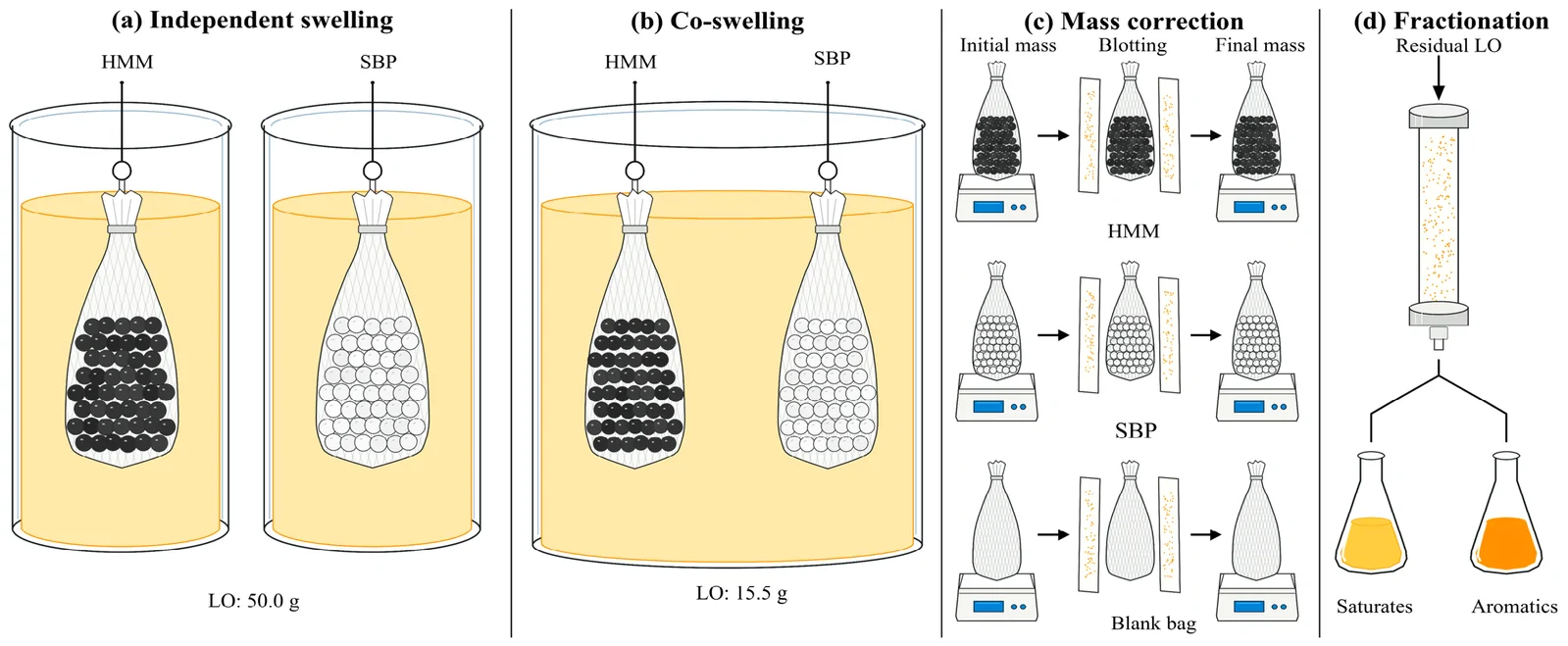
Schematic of the HMM–SBP competitive absorption test and residual light-oil fractionation.

(1) Frequency sweeps were performed using a DSR following ASTM D7175 at 46–70 °C with 25 mm plates and a 1 mm gap. Tests covered 0.1–100 rad/s at a strain amplitude of 0.5%, selected within the LVE region determined by preliminary strain sweeps. Storage modulus (G′), loss modulus (G″), and phase angle (δ) were recorded.
(2) MSCR tests were conducted based on ASTM D7405 at 40–82 °C using unaged binders. Ten creep–recovery cycles were applied sequentially at 0.1 and 3.2 kPa, with 1 s loading and 9 s recovery per cycle. Percent recovery (R) and nonrecoverable creep compliance (Jnr) were determined.
(3) BBR tests were conducted according to ASTM D6648 at −6, −12, and −18 °C under a 980-mN load. Creep stiffness (S) and m-value at 60 s were obtained to characterize low-temperature creep and relaxation behavior.
(4) A modified PTFE mesh-bag method (Figure 1) was used to evaluate competitive light-fraction absorption by HMM and SBP. Based on an equivalent aggregate mass of 500 g, modifier masses were determined from their dry-process dosages (Table 4). With an asphalt-to-aggregate ratio of 5.8% and a combined saturate and aromatic fraction of 53.6% in the base asphalt, 15.5 g of LO was used for co-swelling tests (500 × 5.8% × 53.6% = 15.5 g).

HMM and SBP were separately sealed in 200-mesh PTFE bags. For individual swelling, each modifier was immersed in 50.0 g of excess LO to determine its reference swelling ratio (SRind). For co-swelling, both bags were simultaneously immersed in 15.5 g of limited LO. After conditioning at 160 °C for 60 min, the samples were removed, wiped to remove surface oil, and weighed with blank-bag correction. Each condition was evaluated using 4 independent tests. The swelling ratio (SR) and apparent competition inhibition index of SBP (CI_SBP_) were then calculated. A material-retention test was conducted at 160 °C for 120 min to verify material retention after softening. Residual LO was subsequently fractionated according to ASTM D4124 to determine changes in aromatic and saturate contents.

The SR was calculated using Equation (1):

(1) $$\mathrm{SR}(\%)\;=\;\frac{M_{t}\;-\;M_{0}}{M_{0}}\;\times \;100\%$$

where $M_{0}$ is the initial mass of the modifier before swelling, and $M_{t}$ is the final swelling mass after removing surface free oil and applying a blank-bag correction.

To characterize the overall hindrance of coexisting modifiers on polymer development under a limited LO supply, the CISBP of SBP was defined, as shown in Equation (2):

(2) $${\mathrm{CI}}_{\mathrm{SBP}}\;=\;(1-\frac{{\mathrm{SR}}_{\mathrm{SBP},\mathrm{co}}}{{\mathrm{SR}}_{\mathrm{SBP},\mathrm{ind}}})\;\times \;100\%$$

where (${\mathrm{SR}}_{\mathrm{SBP},\mathrm{co}}$) is the actual swelling ratio of SBP under limited-liquid competitive conditions, and (${\mathrm{SR}}_{\mathrm{SBP},\mathrm{ind}}$) is the individual reference swelling ratio of SBP measured in excess LO.

#### 2.2.4. Molecular Dynamics Simulation

**Figure 2:**
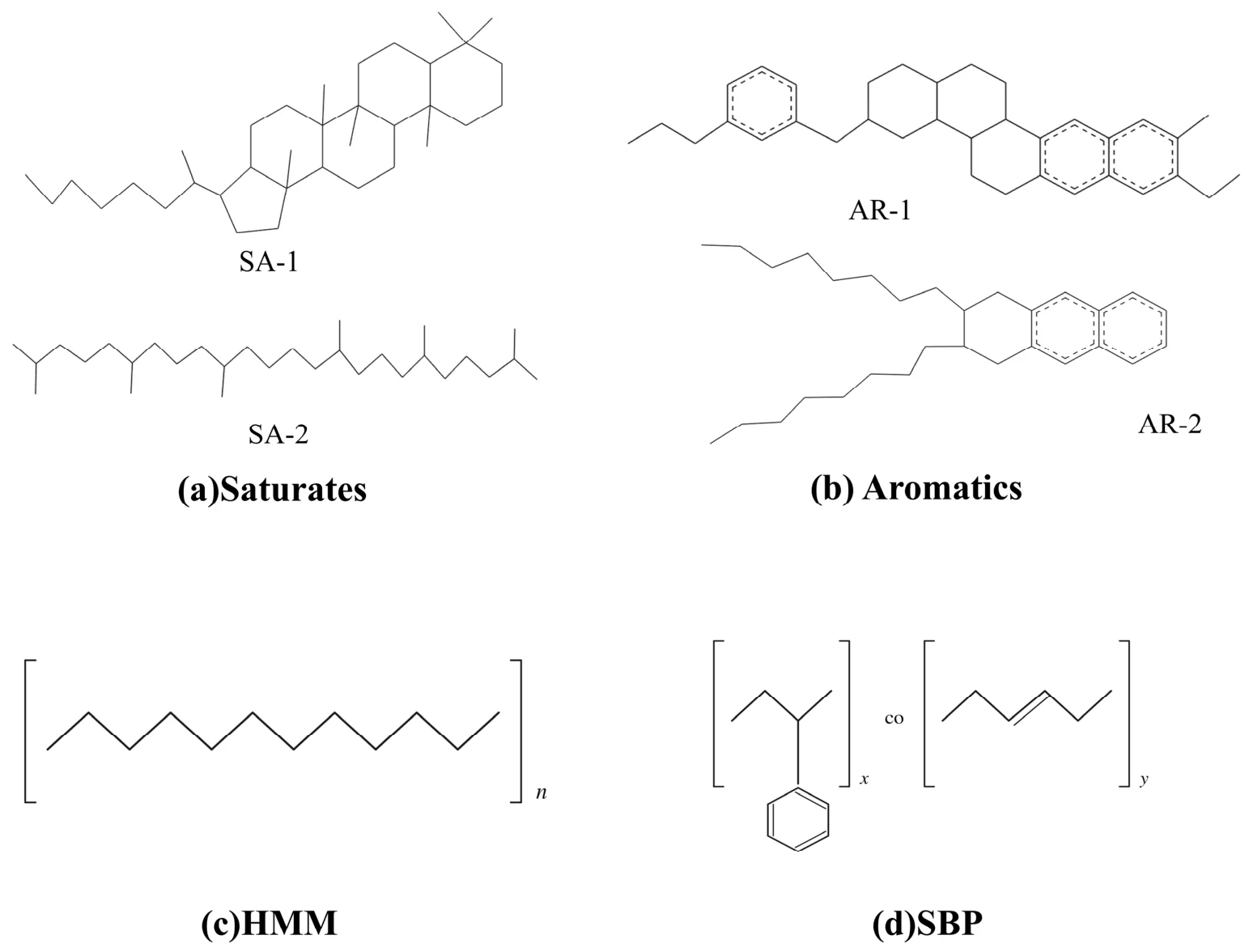
Representative molecular structures used in the molecular dynamics simulations: (**a**) saturates; (**b**) aromatics; (**c**) HMM; and (**d**) SBP.

Molecular dynamics (MD) simulations were performed in Materials Studio 8.0 to compare interactions between HMM, SBP, and the aromatic (AR) and saturate (SA) fractions represented in the LO model (Figure 2). The LO was represented by AR-1, AR-2, SA-1, and SA-2 at a ratio of 11:13:4:4, corresponding to an aromatic-to-saturate mass ratio of 74.13:25.87. HMM was modeled as a polyolefin chain with a degree of polymerization of 80, while SBP contained styrene and butadiene segments at a nominal mass ratio of 75:25. The LO composition was selected to closely match the measured aromatic-to-saturate mass ratio. Finite polymer chains provided computationally tractable representations of the modifier chemistry, with a fixed chain length for each modifier across the systems. Model compositions are summarized in Table 5 [31].

Periodic cells were constructed using the Amorphous Cell module at an initial density of 0.60 g/cm^3^ and simulated using the COMPASS II force field [32]. This initial density was used to facilitate molecular packing, while subsequent NPT simulation allowed volume and density relaxation at the target temperature and pressure [33]. After geometry optimization and annealing between 300 and 600 K for 1000 ps, each system was equilibrated under NPT conditions at 433 K and 1 atm for 500 ps, followed by a 500 ps NVT production run with a 1 fs time step. The target temperature of 433 K corresponds approximately to the experimental swelling temperature of 160 °C. Annealing was used to promote configurational relaxation before NPT relaxation, and the NVT stage provided configurations for interaction-energy sampling at fixed volume and temperature. Temperature and pressure were controlled using the Nosé–Hoover thermostat and Berendsen barostat, respectively. Electrostatic interactions were evaluated using the Ewald method, while van der Waals interactions were calculated using the Atom-based method with a 15.5 Å cutoff. Three independent cells were simulated for each system, and statistics were obtained from configurations sampled during the final 100 ps.

For multicomponent systems, the pairwise interaction energy ($E_{\mathrm{Inter}}(i,j$)) between specific components was calculated using Equation (3):

(3) $$E_{\mathrm{Inter}}(i,j)\;=\;E_{i+j}\;-\;(E_{i}\;+\;E_{j})$$

where i and j denote the selected component groups. E_i+j_ is the potential energy of the combined i − j subsystem, while E_i_ and E_j_ are the potential energies of the individual groups evaluated using the same force field, atomic coordinates, and cell settings, without further relaxation. Keeping the configurations unchanged allows the intracomponent energy contributions to cancel upon subtraction, yielding the interaction contribution between the selected groups. Energy-difference calculations have been used to evaluate polymer–asphalt interactions [34]. Under the sign convention adopted here, negative values indicate net attractive contributions.

To account for differences in modifier abundance, the pair interaction energies were additionally normalized by the number of chains of the corresponding modifier, as expressed in Equation (4):

(4) $$E_{\mathrm{Inter},\mathrm{avg}}\left(p,j\right)\;=\;\frac{E_{\mathrm{Inter}}\left(p,j\right)}{Np}$$

where p denotes HMM or SBP, j denotes the aromatic or saturate fraction, and Np is the number of chains of modifier p in the simulation cell. The normalized values describe the average interaction energy per modifier chain and were used for comparisons involving the same modifier with a fixed chain length across systems.

**Figure 3:**
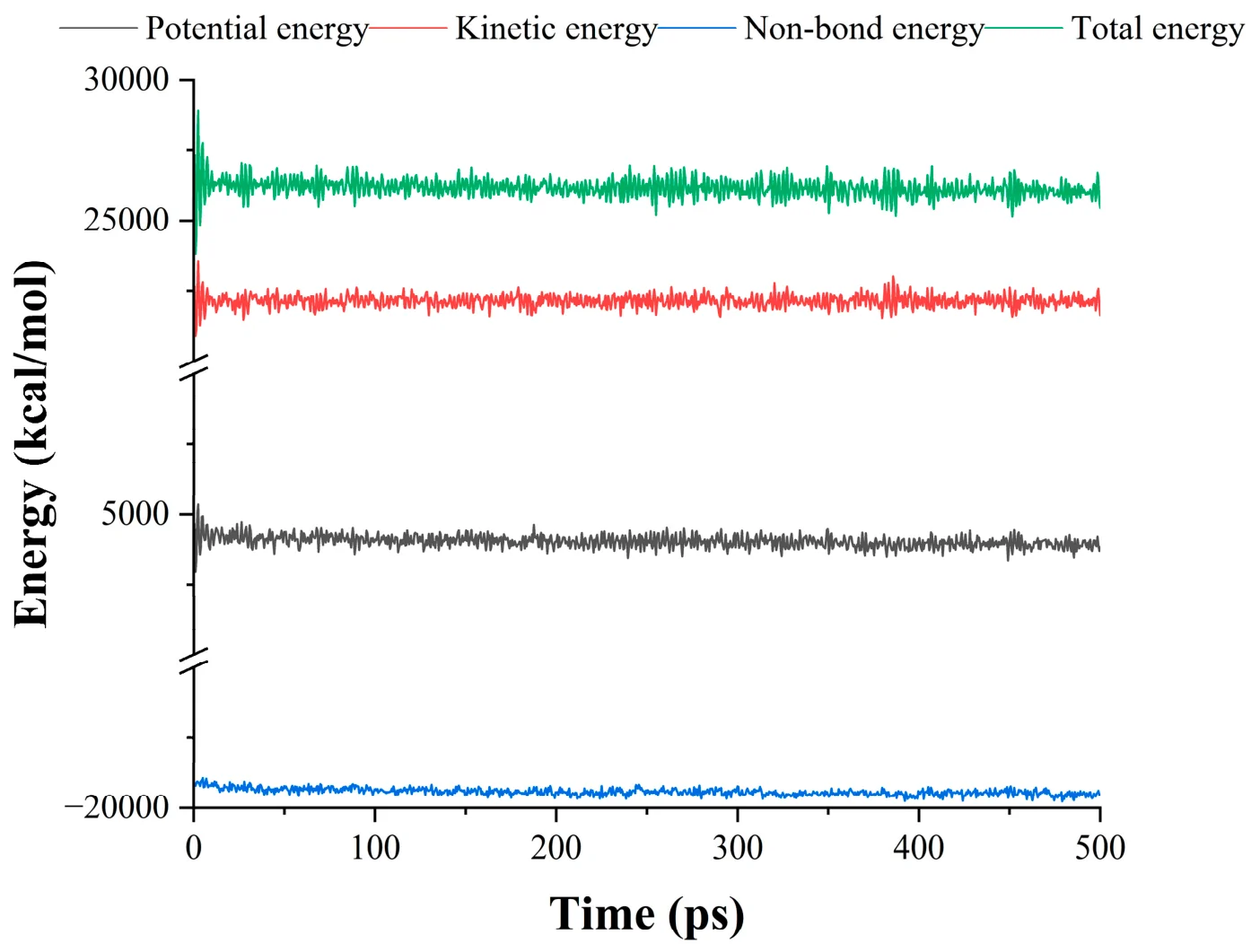
Time evolution of energy components for the H0.6–S0.4–LO system during the NPT simulation.

Figure 3 shows the evolution of potential, kinetic, non-bond, and total energies during the 500-ps NPT relaxation of the H0.6–S0.4–LO system at 433 K and 1 atm. Following the initial adjustment, the energy components fluctuate around relatively stable levels without pronounced systematic drift, supporting energetic stabilization during this stage.

#### 2.2.5. Performance Evaluation of Mixtures

(1) Rutting resistance was evaluated by wheel-tracking tests at 60 °C following JTG 3410–2025 T0719, with dynamic stability (DS) used as the evaluation index.
(2) Low-temperature cracking resistance was assessed by bending tests at −10 °C following JTG 3410–2025 T0715. Beam specimens were conditioned for 4 h and loaded at 50 mm/min to obtain bending strain and strength.
(3) Dynamic modulus and phase angle were measured using an AMPT based on AASHTO T 378. Unconfined cyclic loading was applied at 15 °C over 0.1–25 Hz.
(4) Moisture stability was evaluated using immersion Marshall and freeze–thaw splitting tests according to JTG 3410–2025 T0709 and T0729, respectively, with MSR and TSR as the corresponding indices.

For both the binder and mixture experiments, four independent batches were prepared and tested for each experimental group (*n* = 4). Results are presented as mean ± standard deviation, with error bars representing standard deviations across the four independent batches.

## 3. Results and Discussion

### 3.1. Performance Imbalance of Baseline HMAMs

Four mixtures were evaluated: a control without HMM (H0) and mixtures containing 0.3%, 0.6%, and 1.0% HMM (H0.3, H0.6, and H1.0).

**Figure 4:**
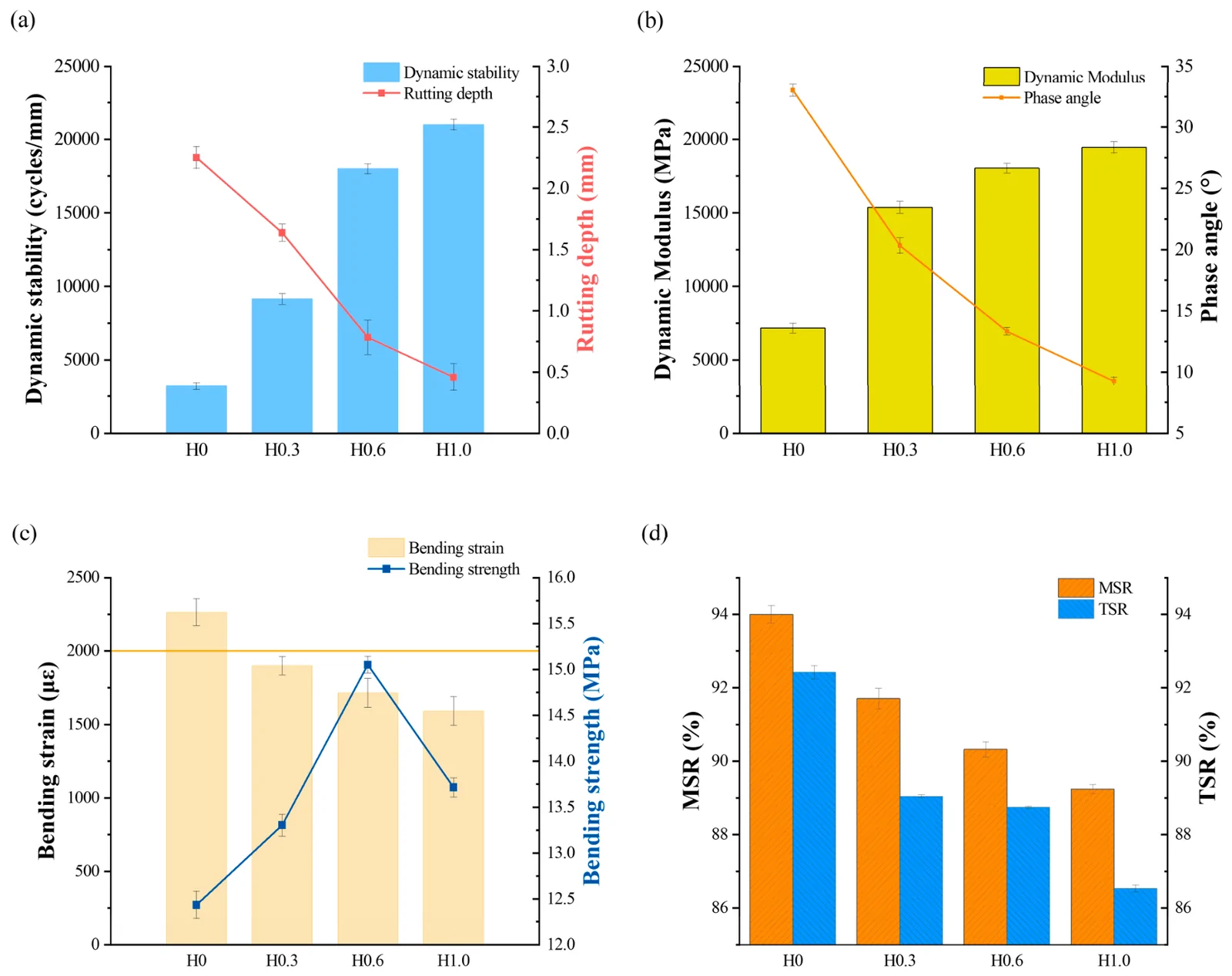
Performance characteristics of baseline HMAMs with varying HMM contents: (**a**) rutting performance; (**b**) dynamic modulus and phase angle at 15 °C and 10 Hz; (**c**) low-temperature bending performance at −10 °C; and (**d**) moisture stability.The solid horizontal line indicates the reference strain limit of 2000 με for the low-temperature bending test.

HMM significantly enhanced high-temperature deformation resistance (Figure 4a). Dynamic stability increased from 3197 cycles/mm for H0 to 21,000 cycles/mm for H1.0, while rut depth decreased from 2.25 to 0.46 mm. Similarly, the dynamic modulus at 15 °C and 10 Hz increased from 7173 MPa to 15,383, 18,037, and 19,454 MPa for the HMM mixtures (Figure 4b). All three exceeded the adopted stiffness benchmark of 14,000 MPa [35]. Meanwhile, the phase angle decreased from 33.02° to 9.26°, indicating a progressively more elastic response. A substantial modulus increase was already achieved at 0.3% HMM, whereas the incremental gains became less pronounced at higher HMM contents.

Conversely, the increase in stiffness reduced low-temperature deformation capacity (Figure 4c). At −10 °C, the bending strain decreased from 2264 με for H0 to 1900, 1715, and 1591 με for H0.3, H0.6, and H1.0, respectively. These values were 16%, 24%, and 30% lower than that of H0 and were all below the 2000 με criterion. Bending strength initially increased from 12.43 MPa for H0 to 13.30 and 15.05 MPa for H0.3 and H0.6, respectively, before decreasing to 13.72 MPa for H1.0. The simultaneous reductions in strength and strain at 1.0% HMM indicated increased low-temperature brittleness. Moisture stability also decreased moderately (Figure 4d), with MSR and TSR declining from 94.0% and 92.4% for H0 to 89.2% and 86.5% for H1.0, respectively, while all mixtures still satisfied the specification requirements (JTG F40–2004).

Although 0.3% HMM met the adopted stiffness benchmark, further increases in HMM content provided diminishing stiffness gains while progressively reducing low-temperature deformation capacity. This stiffness–cracking trade-off is consistent with Chen et al. [6], who reported high rutting resistance but limited cracking resistance in high-modulus asphalt binders.

### 3.2. Effect of SBP Dosage on Low-Temperature Performance

To improve low-temperature cracking resistance, 0.2%, 0.4%, and 0.6% SBP were introduced into the baseline HMAMs, designated as H[X]–S[Y], where X and Y represent the HMM and SBP dosages, respectively.

**Figure 5:**
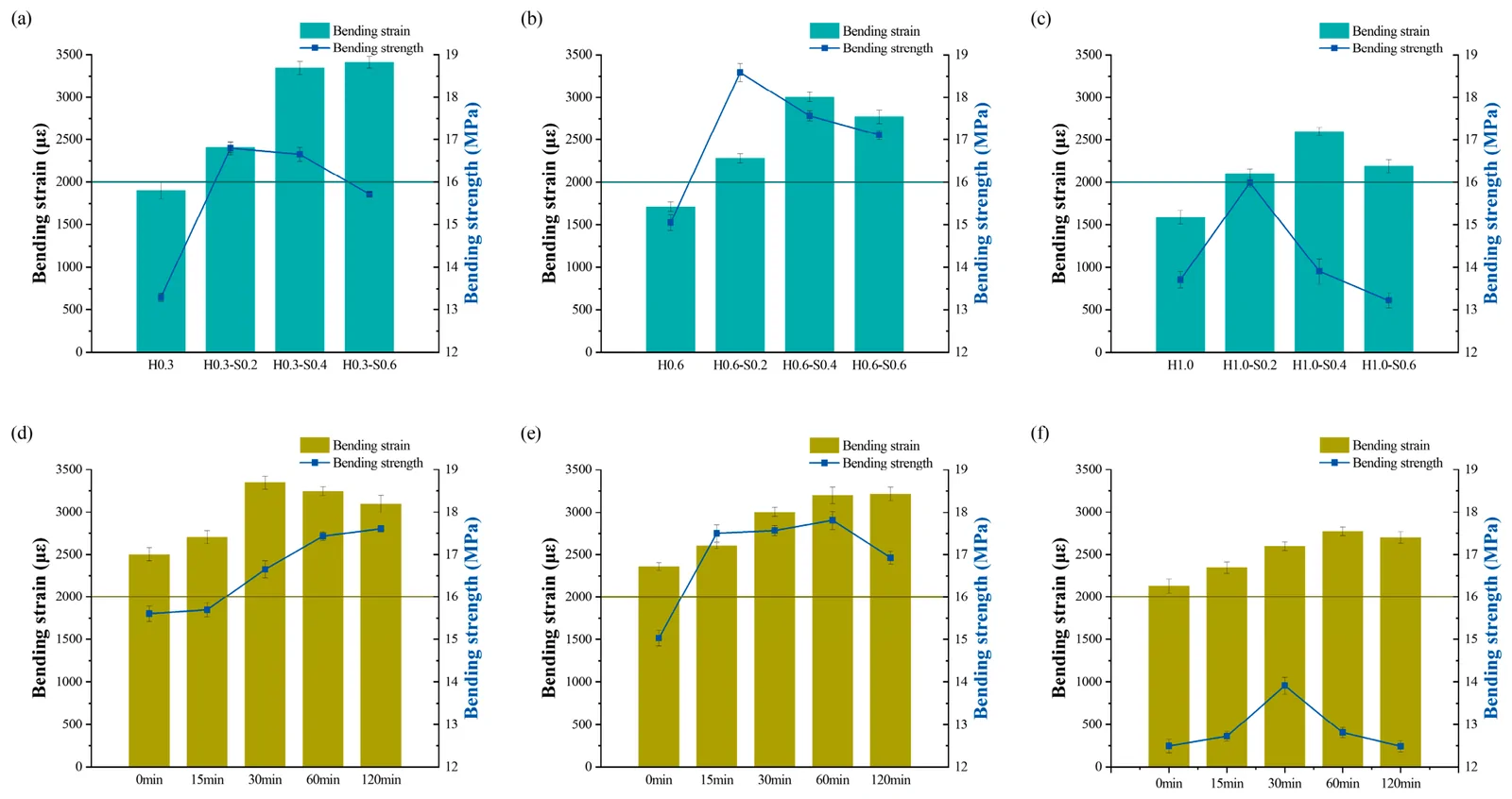
Low-temperature bending responses of HMAMs at different SBP dosages and conditioning times: (**a**,**d**) H0.3; (**b**,**e**) H0.6; and (**c**,**f**) H1.0. Panels (**a**–**c**) show the effect of SBP dosage after 30 min of conditioning, whereas panels (**d**–**f**) show the effect of conditioning time at 0.4% SBP.

For H0.3 (Figure 5a), bending strain increased from 1900 με to 2405, 3346, and 3411 με at 0.2%, 0.4%, and 0.6% SBP, respectively. The 0.4% dosage increased bending strain by 76.1% relative to the baseline, whereas increasing the SBP dosage to 0.6% provided only a marginal additional gain of 1.9% and reduced bending strength. This indicates diminishing toughening benefits at excessive SBP dosages, accompanied by a loss in bending strength.

H0.6 exhibited a more pronounced non-monotonic response to SBP dosage (Figure 5b). Bending strain peaked at 3006 με with 0.4% SBP and then decreased to 2771 με at 0.6%, while bending strength reached a maximum of 18.59 MPa at 0.2% SBP before declining. H1.0 showed the greatest dosage sensitivity (Figure 5c), with bending strain increasing to 2597 με at 0.4% SBP and then decreasing to 2194 με at 0.6%. Meanwhile, bending strength decreased from 15.99 MPa at 0.2% SBP to 13.23 MPa at 0.6%, slightly below that of the untoughened mixture.

Overall, 0.4% SBP provided the most favorable balance between strength and deformation capacity across all HMM systems, increasing bending strain by 76.1%, 75.3%, and 63.2% for H0.3, H0.6, and H1.0, respectively. The progressively smaller relative improvement indicates reduced SBP toughening efficiency at higher HMM contents. A possible explanation is that increased HMM loading limits the availability of mobile light fractions for SBP swelling and subsequent network development.

### 3.3. Effect of Conditioning Time on Low-Temperature Performance

Using 0.4% SBP as the selected dosage, the effect of high-temperature conditioning time on low-temperature toughening was evaluated at 0, 15, 30, 60, and 120 min, with 0 min representing no conditioning. Low-temperature performance showed a nonlinear response, initially improving and subsequently stabilizing or declining.

For H0.3–S0.4, as shown in Figure 5d, bending strain increased from 2502 με at 0 min to a maximum of 3346 με at 30 min, corresponding to an increase of 33.7%. It subsequently decreased to 3247 με at 60 min and 3100 με at 120 min. Bending strength increased from 15.60 to 17.60 MPa over the same period. This suggests that SBP development in the H0.3 system was largely completed within 30 min, while further conditioning provided limited additional benefits. Therefore, 30 min was considered an appropriate conditioning time for H0.3–S0.4.

For H0.6–S0.4, as shown in Figure 5e, bending strain increased from 2361 με at 0 min to 3006 με at 30 min and 3201 με at 60 min, representing an increase of 35.6%. Only a marginal increase to 3218 με occurred at 120 min. Bending strength remained between 17.50 and 17.81 MPa between 15 and 60 min and then decreased to 16.93 MPa, supporting an optimal conditioning time of 60 min. H1.0–S0.4 showed a similar delayed response, as shown in Figure 5f. Bending strain increased from 2130 με at 0 min to 2771 με at 60 min, an increase of 30.1%, before slightly decreasing to 2701 με at 120 min, while bending strength peaked earlier at 30 min.

At 30 min, the relative strain improvement decreased from 33.7% for H0.3 to 27.3% for H0.6 and 21.9% for H1.0, indicating a slower toughening response at higher HMM contents. Higher HMM contents may alter the binder viscoelastic environment and the availability of mobile light fractions, thereby delaying sufficient SBP development [36]. Prolonged conditioning may also cause oxidative hardening or changes in polymer structure, potentially offsetting further benefits from modifier development.

Luo et al. [26] identified incomplete SBS swelling as a limitation of dry modification and investigated accelerated swelling technology. In the present study, the appropriate conditioning time at 0.4% SBP increased from approximately 30 min for H0.3 to approximately 60 min for H0.6 and H1.0, indicating that dry-process conditioning should be tailored to HMM content.

### 3.4. Comprehensive Performance of the Optimized Systems

To determine whether low-temperature toughening compromised other pavement properties, the optimized systems—H0.3–S0.4 conditioned for 30 min and H0.6–S0.4 and H1.0–S0.4 conditioned for 60 min—were compared with their untoughened counterparts. Formulation selection considered retention of the study-adopted stiffness benchmark of |E*| ≥ 14,000 MPa at 15 °C and 10 Hz, improved low-temperature bending strain with maintained strength, and limited reductions in rutting resistance and moisture stability.

**Figure 6:**
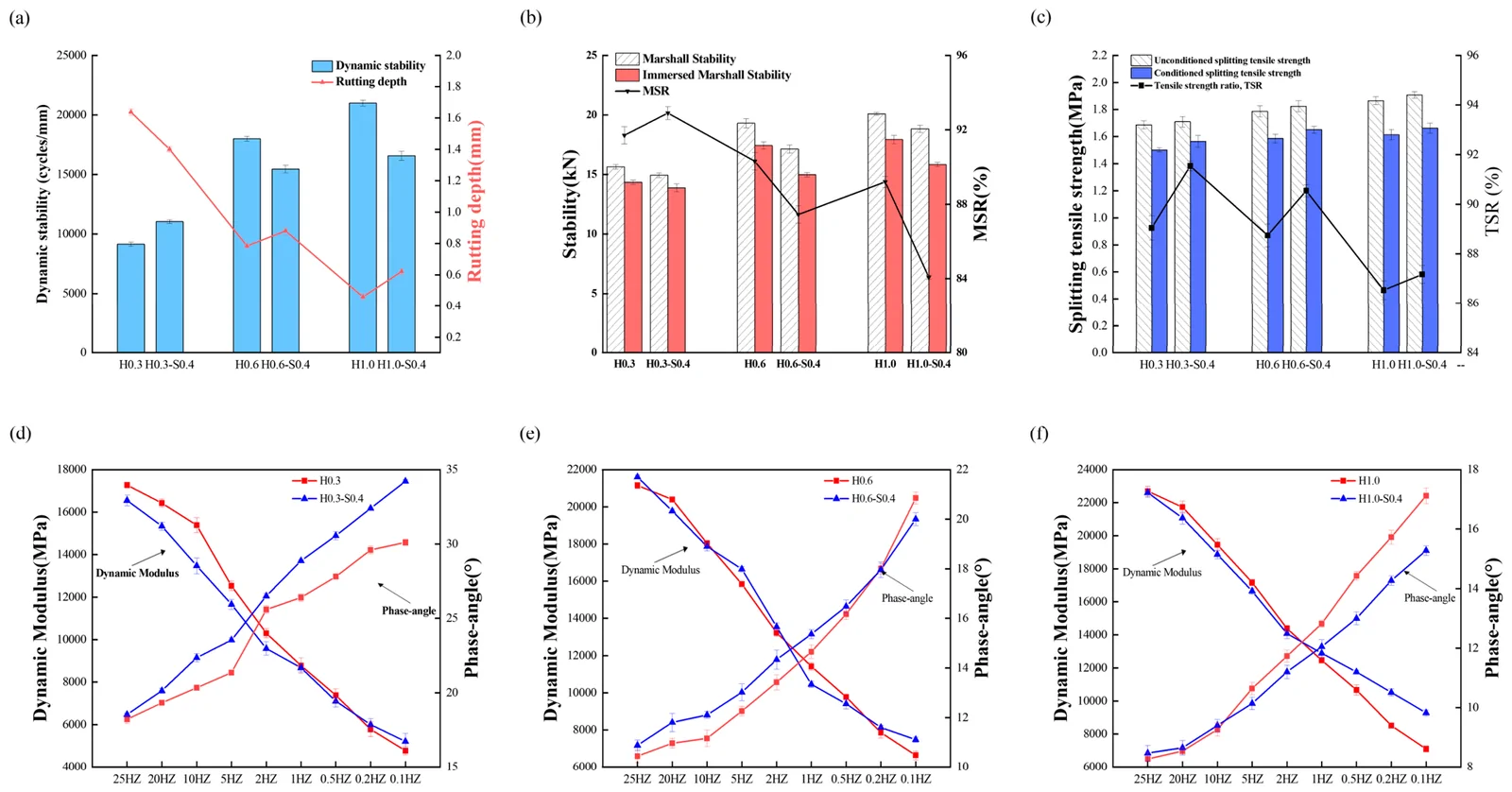
Performance of baseline and optimized SBP-toughened HMAMs: (**a**) dynamic stability and rut depth; (**b**) Marshall stability and retained Marshall stability ratio (MSR); (**c**) splitting tensile strength and tensile strength ratio (TSR); and (**d**–**f**) dynamic modulus and phase angle for the H0.3, H0.6, and H1.0 systems, respectively.

The effect of SBP on rutting resistance depended strongly on HMM content (Figure 6a). For H0.3, SBP increased dynamic stability from 9145 to 11,039 cycles/mm and reduced rut depth by 14.5%. In contrast, the dynamic stability of H0.6–S0.4 and H1.0–S0.4 decreased by 14.1% and 21.1%, while rut depth increased by 12.2% and 35.3%, respectively. Nevertheless, both systems retained higher absolute rutting resistance than H0.3–S0.4.

Moisture stability showed a different response (Figure 6b,c). The MSR of H0.3 increased slightly to 92.90%, whereas those of H0.6–S0.4 and H1.0–S0.4 decreased by 2.86 and 5.12 percentage points, respectively. In contrast, TSR increased by 2.50, 1.80, and 0.64 percentage points for the three systems, although the improvement diminished with increasing HMM content. The greater deformation tolerance provided by SBP may alleviate local stress concentration during freeze–thaw cycling but offers limited benefit under continuous immersion.

The dynamic mechanical responses are shown in Figure 6d–f. The dynamic modulus decreased, and the phase angle increased with decreasing frequency. At 10 Hz, the modulus of H0.3–S0.4 decreased by 12.4% and fell slightly below the adopted stiffness benchmark of 14,000 MPa, whereas H0.6–S0.4 and H1.0–S0.4 showed only minor changes. At 0.1 Hz, however, all toughened systems exhibited higher moduli than their untoughened counterparts, with H1.0–S0.4 increasing by 30.8%. These results indicate that the effect of SBP on dynamic stiffness was strongly frequency-dependent.

H0.3–S0.4 improved low-temperature performance but fell below the adopted stiffness benchmark. H0.6–S0.4 retained this stiffness level and exhibited higher low-temperature bending strain than H1.0–S0.4. Its reductions in dynamic stability and MSR relative to the corresponding untoughened mixture were also smaller: 14.1% and 2.86 percentage points, compared with 21.1% and 5.12 percentage points for H1.0–S0.4. Although H1.0–S0.4 retained higher absolute rutting resistance, H0.6–S0.4 provided a favorable observed balance among the evaluated properties and was therefore recommended within the tested range.

### 3.5. Binder Rheological Response and Competitive Absorption

The rheological results described below characterize the laboratory-prepared model binders and assist qualitative interpretation of the mixture responses. The conditioning requirements of the dry-process mixtures were determined from mixture tests rather than inferred from the model-binder preparation conditions.

#### 3.5.1. Frequency-Dependent Rheological Behavior

**Figure 7:**
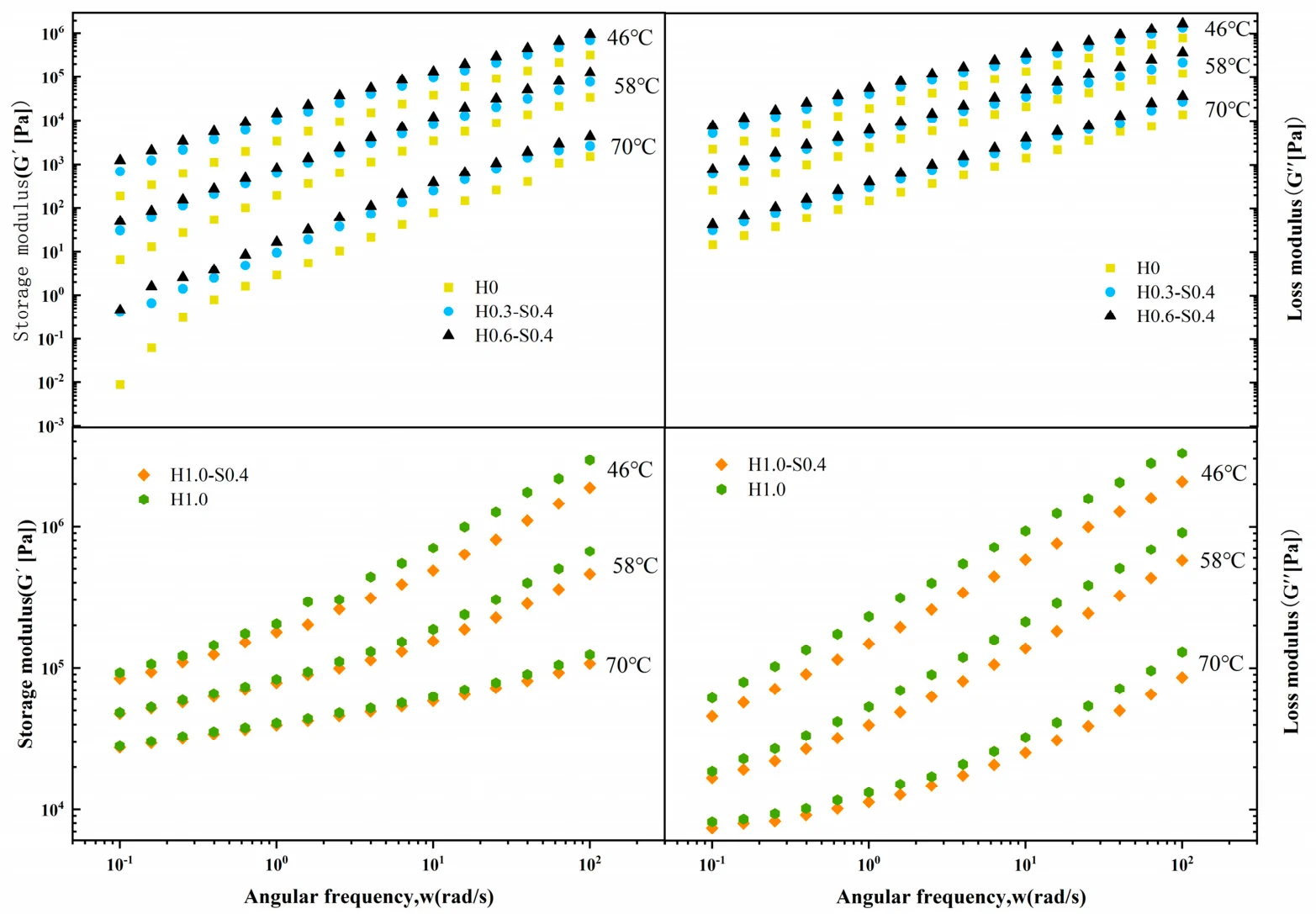
Frequency-dependent rheological responses of different binders at representative temperatures of 46, 58, and 70 °C.

As shown in Figure 7, the storage modulus (G′) and loss modulus (G″) of all binders increased with frequency, reflecting a stronger stiffness response at shorter loading times. Both moduli increased with HMM content, demonstrating the enhanced deformation resistance of the binders. At low and moderate HMM contents, G″ exceeded G′ over most frequencies, reflecting a predominantly viscous response. In contrast, G′ generally exceeded G″ in H1.0 and H1.0–S0.4, showing that a high HMM content shifted the binder toward elastic-dominant behavior. G′ remained higher than G″ even at low frequencies, indicating a persistent elastic-dominant response over longer loading times.

Compared with H1.0, H1.0–S0.4 exhibited lower G′ and G″ but retained the relationship G′ > G″. Thus, SBP reduced the high stiffness without eliminating the elastic response imparted by HMM. This finding agrees with the mixture results: SBP improved low-temperature bending tensile strain while maintaining a relatively high dynamic modulus and rutting resistance. Therefore, the combined modification adjusted the viscoelastic balance rather than simply softening the binder.

#### 3.5.2. Multiple Stress Creep and Recovery Behavior

**Figure 8:**
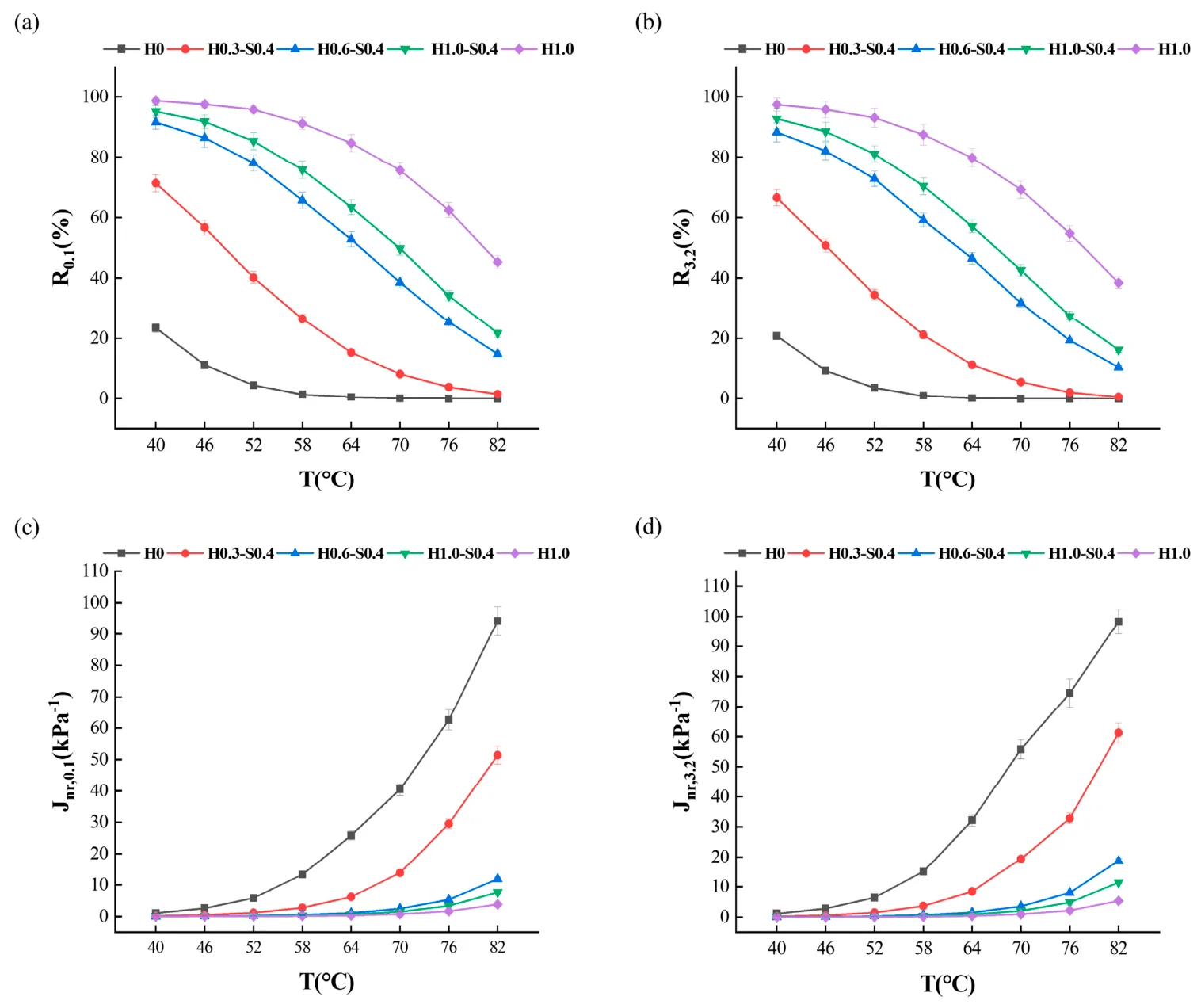
Temperature-dependent MSCR responses of different binders: (**a**) R_0.1_; (**b**) R_3.2_; (**c**) J_nr,0.1_; and (**d**) J_nr,3.2_.

MSCR results at 0.1 and 3.2 kPa are presented in Figure 8. As temperature increased from 40 to 82 °C, percent recovery (R) decreased while nonrecoverable creep compliance (J_nr_) increased, indicating reduced elastic recovery and greater susceptibility to permanent deformation. Higher stress further reduced R and increased J_nr_, reflecting a stronger nonlinear creep response. At 64 °C and 3.2 kPa, R values for H0, H0.3–S0.4, H0.6–S0.4, H1.0–S0.4, and H1.0 were 0.2%, 11.2%, 46.5%, 57.1%, and 79.8%, respectively, with corresponding J_nr_ values of 32.14, 8.46, 1.51, 0.865, and 0.395 kPa^−1^. Thus, increasing HMM content markedly enhanced elastic recovery and resistance to permanent deformation. Compared with H1.0, H1.0–S0.4 showed lower R and higher J_nr_, indicating that SBP partially reduced the high-temperature stiffness and elasticity imparted by HMM. This trend agrees with the frequency-sweep and mixture rutting results. Nevertheless, H1.0–S0.4 remained superior to the lower-HMM systems, demonstrating that substantial high-temperature deformation resistance was retained after SBP toughening.

#### 3.5.3. Low-Temperature Creep and Relaxation Behavior

**Figure 9:**
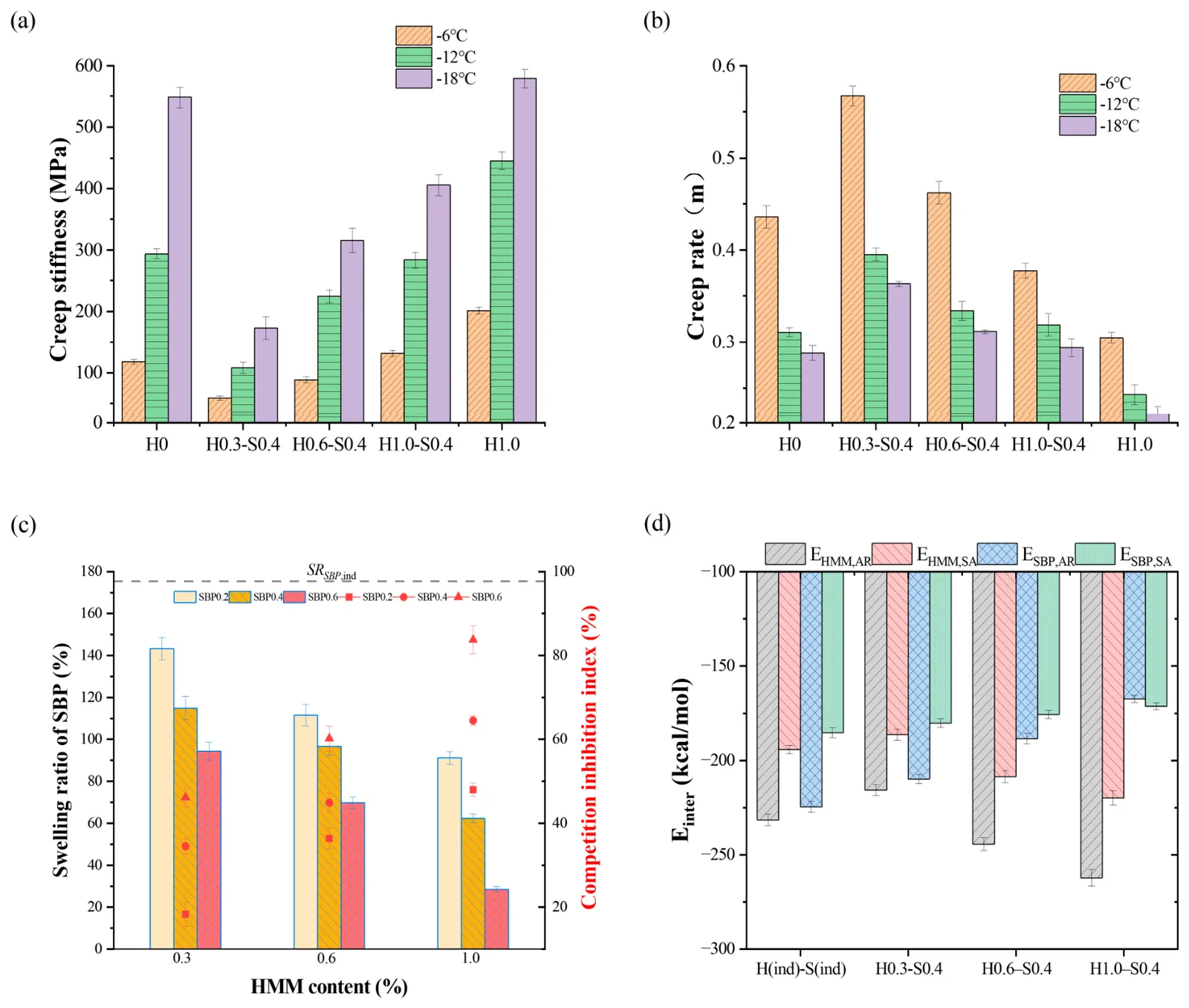
Low-temperature rheological responses and competitive light-fraction absorption in HMM–SBP systems: (**a**) creep stiffness; (**b**) m-value; (**c**) SBP swelling ratio and apparent competition inhibition index; and (**d**) pair interaction energies between the modifiers and light fractions.

BBR tests were conducted to evaluate the low-temperature deformation and stress-relaxation capacities of the binders using the 60-s creep stiffness S and m-value at −6, −12, and −18 °C. As shown in Figure 9a,b, decreasing temperature increased S and reduced the m-value for all binders, reflecting progressive hardening and impaired stress relaxation. The effect of HMM was first evaluated at −12 °C. Compared with H0, H1.0 exhibited an increase in S from 293.5 to 445.6 MPa and a decrease in the m-value from 0.310 to 0.243. This deterioration was consistent with the reduced flexural tensile strain of the baseline mixtures. The effect of SBP was then assessed by comparing H1.0–S0.4 with H1.0. SBP reduced S by 36.5% and increased the m-value by 31.3% at −12 °C. At −18 °C, the corresponding improvements were 30.0% and 33.0%.

Finally, the three binders containing 0.4% SBP were compared at −18 °C. As HMM content increased from 0.3% to 1.0%, S increased from 173.5 to 405.5 MPa, and the m-value decreased from 0.363 to 0.294. Thus, SBP improved low-temperature relaxation but could not fully offset HMM-induced stiffening, possibly because reduced light-fraction availability restricted SBP absorption and swelling.

#### 3.5.4. Competitive Absorption of Light Fractions

To examine whether the HMM-dependent SBP toughening efficiency was associated with competition for limited light fractions, PTFE mesh-bag co-swelling tests were conducted at 160 °C for 60 min. The individual SBP swelling ratio of 175.4% obtained under the same conditions was used as the reference. An apparent competition inhibition index, CI_SBP_, was introduced to quantify the inhibition of SBP swelling under limited-LO conditions.

As shown in Figure 9c, SBP swelling depended strongly on both HMM and SBP dosages. At 0.2% SBP, increasing HMM from 0.3% to 1.0% reduced the swelling ratio from 143.28% to 91.20%, while CI_SBP_ increased from 18.31% to 48.00%. At 0.4% SBP, the swelling ratio decreased from 114.87% for H0.3 to 62.30% for H1.0, with CI_SBP_ reaching 64.48%. The strongest inhibition occurred in H1.0–S0.6, where the swelling ratio decreased to 28.50%, and CI_SBP_ increased to 83.75%. These results indicate that increasing either HMM or SBP dosage intensified the restriction on SBP swelling when the available LO was limited. Under fixed LO supply, the swelling response reflects both modifier loading and relative LO availability. The measured net mass gain may be biased by residual surface oil or modifier loss, and blank-bag correction does not establish a complete mass balance.

Residual LO composition provided further evidence of selective light-fraction absorption, as summarized in Table 6. The initial LO contained 74.13% aromatics and 25.87% saturates, corresponding to an aromatic-to-saturate ratio of 2.87. At 0.4% SBP, increasing HMM from 0.3% to 1.0% reduced the residual aromatic fraction from 65.23% to 48.06% and the corresponding ratio from 1.88 to 0.93. Similarly, at 1.0% HMM, increasing SBP from 0.2% to 0.6% further reduced the residual aromatic fraction from 54.88% to 42.17% and the ratio from 1.22 to 0.73. The preferential depletion of aromatics indicates that these components were more strongly involved in modifier absorption and swelling.

Naderi et al. [30] linked SBS swelling kinetics to bitumen chemical composition. In the present simplified LO system, the reduced SBP swelling at higher HMM loadings suggests that modifier loading relative to the available light fractions is also relevant to polymer swelling. At higher HMM contents, the reduced availability of aromatic components may restrict SBP swelling and development, providing a plausible explanation for the lower toughening efficiency observed in the mixture tests and potentially contributing to the longer conditioning requirement of the higher-HMM systems.

#### 3.5.5. Molecular Interactions Underlying Competitive Absorption of Light Fractions

Figure 9d presents the interaction energies between the modifiers and the aromatic (AR) and saturate (SA) components of LO. All interaction energies were negative, indicating net attractive interactions between the corresponding component groups. With increasing HMM content from H0.3–S0.4–LO to H1.0–S0.4–LO, the HMM–AR and HMM–SA interaction energies changed from −215.68 to −262.17 kcal mol^−1^ and from −186.34 to −219.82 kcal mol^−1^, respectively. The combined HMM–LO interaction-energy magnitude therefore increased by 19.9%. Conversely, the SBP–AR and SBP–SA interaction-energy magnitudes decreased by 20.2% and 4.9%, respectively, resulting in a 13.2% decrease in the combined SBP–LO magnitude. These total-energy changes reflect the combined effects of modifier abundance and the molecular interaction environment.

Table 7 further distinguishes these effects through normalization by the corresponding modifier chain number. The combined HMM–LO interaction-energy magnitude per chain, calculated by summing the normalized AR and SA contributions, decreased from 134.01 to 75.49 and then to 48.20 as HMM content increased. Thus, the increase in total HMM–LO interaction-energy magnitude was accompanied by a decrease in the average contribution per HMM chain and should not be interpreted as stronger interactions per chain. For SBP, the corresponding magnitude decreased from 97.49 to 91.03 and then to 84.66. Because the SBP chain number remained constant across the co-swelling systems, normalization preserved the relative decrease of 13.2%.

Together, these results show that increasing HMM loading altered the modifier–LO interaction environment and reduced the magnitude of the SBP–LO interaction energy at a fixed SBP chain number. This trend is consistent with the reduced SBP swelling observed in the co-swelling tests and provides molecular-level evidence compatible with competition for limited light fractions. Within the simplified LO model, the combined findings suggest that increasing HMM content may limit the availability of light fractions for SBP swelling.

The simplified LO system represents interactions involving aromatics and saturates, while the effects of resins and asphaltenes on component distribution and modifier swelling are not captured. The findings therefore provide model-based support for competitive light-fraction absorption as a possible contributing mechanism. Further studies using actual asphalt binders and molecular models incorporating all four fractions could clarify how the complete binder composition influences this behavior.

The findings were obtained using one base asphalt binder, one HMM, and one SBP type. The selected SBP dosage and conditioning times therefore apply to the investigated material systems. For other binder–modifier combinations, differences in binder composition and modifier characteristics may affect swelling behavior and toughening efficiency, requiring corresponding adjustments to dosage and conditioning time. The integrated evaluation approach adopted here provides a useful framework for assessing these effects and examining the role of competitive light-fraction absorption in other systems.

Because long-term aging was not evaluated in this study, future work should assess the effects of oxidative aging and environmental exposure on the retention of the observed performance improvements. Intermediate-temperature fatigue testing is also needed to assess resistance to repeated loading and determine whether the selected modifier dosages and conditioning times provide a favorable balance among rutting, fatigue, and low-temperature cracking performance.

## 4. Conclusions

This study addressed the stiffness–cracking trade-off of high-modulus asphalt mixtures (HMAMs) by introducing dry-process SBP toughening and examining its rheological and competitive light-fraction absorption mechanisms. The main conclusions are as follows:

(1) SBP exhibited a clear dosage-dependent toughening effect. At 30 min of conditioning, 0.4% SBP increased the low-temperature flexural strain of H0.3, H0.6, and H1.0 by 76.1%, 75.3%, and 63.2%, respectively. Increasing the SBP dosage to 0.6% provided little additional improvement in bending strain for H0.3, whereas both bending strength and strain decreased in the H0.6 and H1.0 systems.
(2) The conditioning requirement depended on HMM content. H0.3–S0.4 reached an effective toughening state at approximately 30 min, whereas H0.6–S0.4 and H1.0–S0.4 required about 60 min. H0.6–S0.4 showed the best overall balance among low-temperature deformability, dynamic modulus, rutting resistance, and moisture stability, indicating that conditioning time should be tailored to modifier composition rather than fixed.
(3) SBP modified rather than simply softened the viscoelastic response of high-modulus binders. For H1.0, 0.4% SBP reduced creep stiffness by 36.5% and increased the m-value by 31.3% at −12 °C, mitigating HMM-induced low-temperature stiffening while retaining substantial high-temperature elastic recovery and deformation resistance.
(4) Co-swelling tests and molecular simulations provided evidence consistent with competitive absorption under limited-LO conditions. Increasing HMM from 0.3% to 1.0% reduced the swelling ratio of 0.4% SBP from 114.87% to 62.30%, accompanied by a decrease in the aromatic proportion of the residual LO. The SBP–LO interaction-energy magnitude per model chain decreased by 13.2% as HMM content increased. Together, these findings suggest that competition for available light fractions may restrict SBP swelling and contribute to the HMM-dependent toughening efficiency and conditioning requirements.

## Figures and Tables

**Table 1:** Physical properties of 70-penetration-grade base asphalt.

Property	Test Method	Value
Physical Properties		
Penetration (25 °C, 0.1 mm)	ASTM D5	68.3
Ductility (15 °C, cm)	ASTM D113	>100
Softening point (°C)	ASTM D36	48.3
Density (15 °C, g/cm^3^)	ASTM D70	0.997
Chemical Composition (wt%)		
Saturates	ASTM D4124	13.5
Aromatics		40.1
Resins		27.2
Asphaltenes		19.2

**Table 2:** Aggregate gradation for HMAC-16.

Sieve Size (mm)	Percentage Passing (%)										
	19	16	13.2	9.5	4.75	2.36	1.18	0.6	0.3	0.15	0.075
Design gradation	100	98.8	89	75	52	36	26.2	20.4	14.4	10.4	7.5
Upper limit	100	100	96	84	64	44	32	25	20	14	8
Lower limit	100	95	82	66	44	30	20	15	11	8	6

**Table 3:** Conversion between dry-process dosages and theoretical equivalent binder dosages.

Material Type	Dry Process Dosage (wt% of Aggregate)	Equivalent Dosage (wt% of Base Asphalt)
HMM	0	0
	0.3	5.2
	0.6	10.3
	1.0	17.2
SBP	0	0
	0.2	3.4
	0.4	6.9
	0.6	10.3

**Table 4:** Material quantities used in individual and co-swelling tests.

Swelling Condition	Modifier Combination	Material	Modifier Mass (g)	LO Mass (g)
Individual swelling	Single Material	HMM	2	50
		SBP	2	
Co-swelling (H0.3)	0.3% HMM + 0.2%SBP	HMM	1.5	15.5
		SBP	1	
	0.3% HMM + 0.4%SBP	HMM	1.5	15.5
		SBP	2	
	0.3% HMM + 0.6%SBP	HMM	1.5	15.5
		SBP	3	
Co-swelling (H0.6)	0.6% HMM + 0.2%SBP	HMM	3	15.5
		SBP	1	
	0.6% HMM + 0.4%SBP	HMM	3	15.5
		SBP	2	
	0.6% HMM + 0.6%SBP	HMM	3	15.5
		SBP	3	
Co-swelling (H1.0)	1.0% HMM + 0.2%SBP	HMM	5	15.5
		SBP	1	
	1.0% HMM + 0.4%SBP	HMM	5	15.5
		SBP	2	
	1.0% HMM + 0.6%SBP	HMM	5	15.5
		SBP	3	

**Table 5:** Molecular definitions and target compositions of the MD systems.

**(a) Molecular Definitions**				
**Code**	**Model Formula**		**Approx. Molar Mass (g/mol)**	
AR-1	C_35_H_44_		464.7	
AR-2	C_30_H_46_		406.7	
SA-1	C_35_H_62_		482.9	
SA-2	C_30_H_62_		422.8	
HMM	C_160_H_322_		2246.3	
SBP	C_168_H_190_		2209.4	
Note: The listed molar masses refer to the finite model chains and do not represent the molecular-weight distributions of the commercial modifiers.				
**(b) MD Systems for Competitive Absorption Analysis**				
**MD System**	**Target HMM:SBP:LO Mass Ratio**	**Number of Chains/Molecules (HMM/SBP/AR-1/AR-2/SA-1/SA-2)**		**Pair Interactions Evaluated**
HMM–LO	2:0:50	2/0/88/104/32/32		$E_{\mathrm{HMM},\mathrm{AR}}$, $E_{\mathrm{HMM},\mathrm{SA}}$
SBP–LO	0:2:50	0/2/88/104/32/32		$E_{\mathrm{SBP},\mathrm{AR}},\;E_{\mathrm{SBP},\mathrm{SA}}$
H0.3–S0.4–LO	1.5:2:15.5	3/4/55/65/20/20		$E_{\mathrm{HMM},\mathrm{AR}}$,${\;E}_{\mathrm{HMM},\mathrm{SA}}$,${\;E}_{\mathrm{SBP},\mathrm{AR}},{\;E}_{\mathrm{SBP},\mathrm{SA}}$
H0.6–S0.4–LO	3:2:15.5	6/4/55/65/20/20		$E_{\mathrm{HMM},\mathrm{AR}}$, $E_{\mathrm{HMM},\mathrm{SA}}$, $E_{\mathrm{SBP},\mathrm{AR}},{\;E}_{\mathrm{SBP},\mathrm{SA}}$
H1.0–S0.4–LO	5:2:15.5	10/4/55/65/20/20		$E_{\mathrm{HMM},\mathrm{AR}}$,${\;E}_{\mathrm{HMM},\mathrm{SA}}$,${\;E}_{\mathrm{SBP},\mathrm{AR}},\;E_{\mathrm{SBP},\mathrm{SA}}$

**Table 6:** Compositional changes of residual light oil after co-swelling.

Test Group	Aromatics/%	Saturates/%	Aro/Sat Ratio
Initial LO	74.13	25.87	2.87
0.3% HMM + 0.2% SBP	69.51	30.49	2.28
0.3% HMM + 0.4% SBP	65.23	34.77	1.88
0.3% HMM + 0.6% SBP	61.87	38.13	1.62
0.6% HMM + 0.2% SBP	63.44	36.56	1.74
0.6% HMM + 0.4% SBP	57.91	42.09	1.38
0.6% HMM + 0.6% SBP	54.12	45.88	1.18
1.0% HMM + 0.2% SBP	54.88	45.12	1.22
1.0% HMM + 0.4% SBP	48.06	51.94	0.93
1.0% HMM + 0.6% SBP	42.17	57.83	0.73

**Table 7:** Pair interaction energies normalized by modifier chain number in the individual and co-swelling model systems (kcal mol^−1^ per model chain).

MD System	HMM–AR	HMM–SA	SBP–AR	SBP–SA
HMM–LO	−115.71	−97.13	—	—
SBP–LO	—	—	−112.28	−92.66
H0.3–S0.4–LO	−71.89	−62.11	−52.46	−45.03
H0.6–S0.4–LO	−40.73	−34.76	−47.12	−43.91
H1.0–S0.4–LO	−26.22	−21.98	−41.84	−42.82

Note: Values are expressed in kcal mol^−1^ and normalized by the number of chains of the corresponding modifier according to Equation (4). A dash indicates that the modifier is absent from the system.

## Data Availability

Some or all data, models, or code that support the findings of this study are available from the corresponding author upon reasonable request.
